# Prevalence of Intestinal Parasites in Dog Faecal Samples from Public Environments in Qinghai Province, China

**DOI:** 10.3390/pathogens11111240

**Published:** 2022-10-26

**Authors:** Xueyong Zhang, Yingna Jian, Yijuan Ma, Zhi Li, Yong Fu, Zhouzai Cairang, Xiaohong Wang, Hong Duo, Zhihong Guo

**Affiliations:** 1 Qinghai Academy of Animal Sciences and Veterinary Medicine, Qinghai University, Xining 810016, China; 2 State Key Laboratory of Veterinary Etiological Biology, Lanzhou Veterinary Research Institute, Chinese Academy of Agricultural Sciences, Lanzhou 730046, China; 3 Gangcha County Animal Husbandry and Veterinary Station, Haibei 812399, China; 4 Guinan County Animal Husbandry and Veterinary Station, Hainan 813100, China

**Keywords:** intestinal parasites, dogs, prevalence, public environments, Qinghai

## Abstract

Dogs are popular companions in our daily lives for company, hunting, protection or shepherding, but they also serve as reservoirs for zoonotic parasites. We analysed faecal samples from urban and rural environments in Qinghai Province on the Qinghai-Tibet Plateau of China to determine the prevalence of intestinal parasites. A total of 682 faecal samples were collected from four urban and two rural environments from October 2019 to December 2020. The samples were analysed for common intestinal parasites using a species-specific PCR approach. The total number of samples with parasites was 40 (5.87%): 23 (3.37%) were positive for helminths, and 17 (2.49%) were positive for protozoa. The following parasites were identified, and their respective prevalence rates were calculated: *Cryptosporidium canis* (1.32%), *Giardia duodenalis* (1.17%, assemblages D (*n* = 6) and C (*n* = 2)), *Taenia hydatigena* (1.03%), *Taenia multiceps* (0.59%), *Toxocara canis* (0.59%), *Echinococcus shiquicus* (0.29%), *Dipylidium caninum* (0.29%), *Taenia pisiformis* (0.15%), *Mesocestoides lineatus* (0.15%), *Trichuris vulpis* (0.15%), and *Ancylostoma* spp. (0.15%). The overall prevalence was significantly higher in dog faecal samples from rural environments than in those from urban environments (16.19% vs. 3.99%). *E. shiquicus*, *T. pisiformis*, *M. lineatus*, *T. vulpis*, and *Ancylostoma* spp. were only found in dog faecal samples from rural environments. The results of the present study indicate that intestinal parasite-positive dogs are important sources of environmental contamination, suggesting a significant zoonotic infection risk in humans and other animals. This has implications for the ongoing control of intestinal parasite infections in dogs in Qinghai Province, China.

## 1. Introduction

Dogs have close relationships with humans in everyday life. In urban areas, dogs are often kept as pets for companionship, and in rural areas, such as pastoral areas, dogs aid in hunting and protection. However, dogs may serve as reservoirs for zoonotic gastrointestinal parasites, which are of significant public health concern worldwide [1,2]. Dogs usually act as definitive hosts and contribute to the transmission of zoonotic infections by shedding large numbers of eggs of infective helminths (*Taenia hydatigena*, *Taenia multiceps*, *Dipylidium caninum*, *Echinococcus* spp., *Taenia pisiformis*, *Mesocestoides lineatus*, *Trichuris vulpis*, *Toxocara canis*, *Toxascaris leonine*, *Spirocerca lupi*, *Clonorhis sinensis*, *Spirometra mansoni*, *Strongyloides* spp., and *Ancylostoma* spp.) and oocysts (cysts) of protozoans (*Giardia* spp., *Cryptosporidium* spp., *Cystoisospora* spp., and *Neospora* spp.) in faeces, which contaminate the environment [3,4,5].

In most cities in China, the government requires that dogs be registered, tagged and kept on leashes or ropes. Information about the risks of zoonotic diseases transmitted by dogs is practically nonexistent. In urban areas, dog owners do not proactively clean up their dogs’ faeces, and this faecal contamination could lead to an increased risk of exposure to zoonoses [6]. Indeed, owners often bring their dogs to parks, squares and green paths where the dogs can excrete freely, but only a few responsible owners clean up after them. In rural areas, unrestrained dogs, such as stray and farm dogs, wander freely, sometimes live in close contact with livestock, and often have access to human environments (herder tents), representing a potential risk to public and animal health. In Qinghai Province, many epidemiological studies on *Echinococcus* spp. prevalence in the canine population have been conducted [7,8,9]. In other areas, some studies on intestinal parasites in dogs have been conducted. For instance, in a previous study in Beijing, China, among outpatient pet dogs with diarrhoea, 25.6% harboured one or more parasites [10]. In Heilongjiang Province of north-eastern China, a total of 178 adult farm dogs were examined, and all were infected with more than one helminth species [11]. These results were similar to those of a study performed in Hunan Province, China, which found that a total of 438 adult farm dogs were infected with at least one helminth species [12]. In Guangzhou city in southern China, it was reported that stray and shelter dog faecal samples screened by light microscopy had an overall helminth prevalence of 29.53% [13]. Another recent study was carried out in Guangdong Province, China, and the results showed that the prevalence of hookworms in stray dogs was 20.23% [14]. In Guangxi, a province in southern China, gastrointestinal helminths were found in all necropsied dogs (*n* = 40) [4].

Although few studies have been conducted in some parts of China, intestinal parasites, including helminths, hookworms and protozoans, appear to be common in dogs. There are few investigations on the gastrointestinal parasites present in public environments (parks, squares and green paths), where the copresence of dogs, as zoonotic reservoirs, could increase the risks for human infections, since deworming and the identification and prevention of parasite infections are often ignored. Therefore, the aim of the present study was to determine the prevalence of canine intestinal parasites in dog faeces in public environments in Qinghai Province, China.

## 2. Materials and Methods

**Figure 1:**
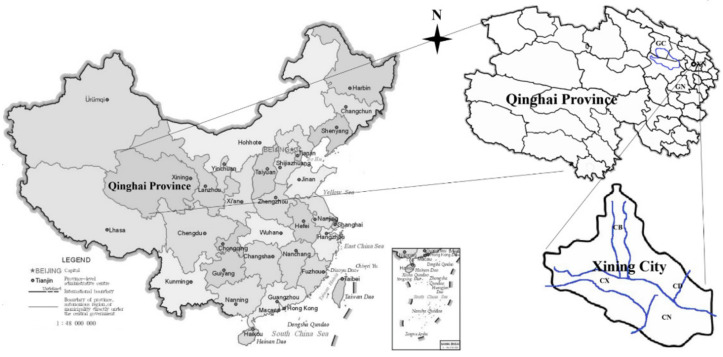
Distribution of the locations of sample collection in this study. Qinghai Province is located on the Qinghai-Tibet Plateau in China. The letter represents the sampling site (sampling site names shown in Table 1). Note: XN: Xining City, CD: Chengdong, CX: Chengxi, CN: Chengnan, CB: Chengbei, GC: Gangcha, GN: Guinan.

Faecal samples were collected from dogs in different locations (parks, squares and green paths) in Qinghai Province, China (Figure 1 and Table 1). The Chengdong area, Chengxi area, Chengnan area and Chengbei area, which are urban environments, are within Xining city, the capital region. Gangcha County and Guinan County are county regions and rural environments. A total of 682 dog faecal samples were collected from the urban environments (577 samples) and rural environments (105 samples). The faecal samples were refrigerated (4 °C), transported to our laboratory and stored (−70 °C) in our laboratory facility until analysis. In the laboratory, total genomic DNA was extracted from each faecal sample with a TIANamp Stool DNA Kit (TIANGEN, Beijing, China) according to the manufacturer’s instructions. Subsequently, faecal DNA samples were amplified using parasite species-specific PCR, as described previously (Supplementary Materials: Table S1). To identify contamination, negative controls (without DNA template) were run with each set of amplification reactions. Positive PCR products were sequenced by TIANJIN GENEWIZ Company (Tianjin, China), and species was confirmed by BLAST (https://blast.ncbi.nlm.nih.gov/Blast.cgi (accessed on 20 August 2022) alignment with reference sequences in GenBank. Prevalence data were compared with the chi-square test (χ^2^) with a significance level of 95% (*p* < 0.05) by using online software (http://www.quantpsy.org/chisq/chisq.htm (accessed on 20 August 2022). Statistical analysis was carried out using Open Source Epidemiologic Statistics Software (http://www.openepi.com/Proportion/Proportion.htm (accessed on 20 August 2022).

## 3. Results

The results of the prevalence rates of the different gastrointestinal parasites found in dog faecal samples from urban and rural environments are shown in Table 1 and Table 2. Intestinal parasites were found in 40 canine faecal samples, with an overall prevalence of 5.87% (95% CI 4.34–7.89%); the prevalence of helminths was 3.37% (95% CI 2.26–5.01), and that of protozoa was 2.49% (95% CI 1.56–4.00). Additionally, there were no mixed infections in any of the dog faecal samples. In total, the most frequently observed intestinal parasite in this study was *Cryptosporidium* spp. (1.32%), followed by *Giardia duodenalis* (1.17%) and *T. hydatigena* (1.03%). The prevalence rates of infections with helminths (*Echinococcus shiquicus*, *T. multiceps*, *D. caninum*, *T. pisiformis*, *M. lineatus*, *T. vulpis*, *T. canis* and *Ancylostoma* spp.) were low and varied from 0.59% to 0.15%. It is worth noting that many parasites (*E. multilocularis*, *E. granulosus*, *T. leonine*, *S. lupi*, *C. sinensis*, *S. mansoni*, *Strongyloides* spp., *Cystoisospora* spp. and *Neospora* spp.) were not detected in dog faecal samples. The positive PCR products were sequenced by forward and reverse primers, and then the obtained sequences were submitted to GenBank and given accession numbers OP654955-OP654972, OP619942-OP619951, OP620561-OP620562, OP620000, OP619961.

When the prevalence of intestinal parasites was analysed by environment type, the results showed that dogs in rural environments (16.19%) had higher rates of infection than those in urban environments (3.99%). There was a significant difference in the overall prevalence of intestinal parasites between dogs from rural environments and urban environments (*p* < 0.01) (Table 2). Similarly, for each individual intestinal parasite infection in dogs, the prevalence was higher in rural environments than in urban environments. Protozoan infections (*n* = 13) were more frequent than helminthic infections in urban environments.

## 4. Discussion

In this study, the prevalence rates of intestinal parasite infections in dogs in China were reported. The overall prevalence in dogs in both environments was low, the prevalence of each parasite species in both environments was low, and some parasites were not detected. Why the prevalence was lower than those in the above-mentioned studies is unclear. The Qinghai-Tibet Plateau region is a hydatid disease-endemic area; in particular, Qinghai Province is a highly endemic area [8,15]. Dogs are the primary definitive hosts in the transmission cycles of *E. multilocularis*, *E. granulosus* and *E. shiquicus*. Periodic deworming of dogs to protect against echinococcosis is required by the Chinese government, and a monthly deworming programme (each dog is dewormed every month) has been implemented to control the transmission of canine echinococcosis. Consequently, significant progress has been made in reducing the infection rate of *Echinococcus* spp. in dogs [16]. Due to the deworming programme, which recommends praziquantel administration, initiated 10 years prior, the *E. multilocularis* prevalence in dogs was significantly (*p* < 0.01) reduced from 7.23% (25/346) in 2000–2003 to 0.55% (1/181) in 2016 in all three evaluated Tibetan communities in Sichuan Province, China [17]. These results suggest that the anthelmintic drug praziquantel is very effective in killing tapeworms, flukes and other intestinal parasites. Nonetheless, because of nomadic production factors and a lack of deworming awareness, it has been difficult to implement manual monthly deworming in every dog. Another study confirmed that some farm dogs in Qinghai Province, where the prevalence of taeniid cestodes was reduced to 9.6% and 4.9% after one- and two- years of implementation of the deworming programme, respectively, were not appropriately receiving praziquantel [9]. In this study, we detected some species of tapeworms (*E. shiquicus*: 0.29%, *T. hydatigena*: 1.03%, *T. multiceps*: 0.59%, *D. caninum*: 0.29%, *T. pisiformis*: 0.15% and *M. lineatus*: 0.15%) with low prevalence rates in dogs in the different environments.

The cestodes *E. multilocularis* and *E. granulosus* were not detected in samples from either urban or rural environments, while *E. shiquicus* was detected in dog faecal samples from rural environments, with a 1.90% prevalence rate. In accordance with the results of our study, similar prevalence rates of *E. shiquicus* were reported in stray dogs (0.7%) in the Golog, Yushu, and Haixi prefectures of Qinghai Province [8]. In the present study, the prevalence rates of *T. hydatigena* were 0.69% and 2.86% in urban and rural environments, respectively, which was lower than that (19.7%) in farm dogs in Heilongjiang Province [11]. The prevalence rates of *T. multiceps* infection were 0.35% and 1.90% in urban and rural environments, respectively, which was lower than that (15.33%) in farm dogs in Hebei Province [18]. *D. caninum* was detected in only one dog each in the urban (0.17%) and rural environments (0.95%); the prevalence rates were lower than those in farm dogs in Heilongjiang (14.6%) [11] and Hunan Provinces (42.3%) [12] and was similar to that in pet dogs in Beijing (0.2%) [10]. *T. pisiformis* infection in dogs was observed in only rural environments, with a 0.95% prevalence rate, which was similar to that in Heilongjiang (1.1%) [11] but lower than that in Huanan (12.9%) [12]. *M. lineatus* was also detected in samples from only rural environments, with a 0.95% prevalence rate, which was lower than that in farm dogs in Heilongjiang (20.2%) [11].

Nematode infections were significantly less frequently detected in urban environments than in rural environments (Table 1 and Table 2). The overall frequency of *T. canis* infection was 0.59% (urban environments: 0.52%, rural environments: 0.95%) in this study, which was comparable to those in previous studies performed in China (45.2% in farm dogs (Hunan) [12], 36.5% in farm dogs (Heilongjiang) [11] and 3.5% in pet dogs (Beijing) [10]). These data indicated an obviously different prevalence between urban environments (Beijing) and rural environments (Hunan and Heilongjiang). The low prevalence (0.15%) of *Ancylostoma* spp. in this study, especially in rural environments (0.95%), was different from the research findings in farm dogs in Hunan (20.3%) [12] and Heilongjiang (66.3%) [11]. *T. vulpis* was also found in only rural environments (0.95%), which was in agreement with findings in pet dogs (0.6%) in Beijing [10]. These results suggest that these parasites could be widely distributed in farms and fields in rural environments. With improvements in hygienic conditions in urban environments, intestinal parasites in the environment have been eliminated. Additionally, intestinal parasites cannot complete their life cycles in urban environments due to a lack of hosts.

Protozoa accounted for the majority of parasites detected in the present study. However, *Cystoisospora* spp. and *Neospora* spp. were not detected, while the prevalence rates of *Cryptosporidium* spp. (1.32%) and *G. duodenalis* (1.17%) were higher than those of other parasites detected in the dogs. When compared with those in previous regional studies, the prevalence of *Cryptosporidium* spp. was lower than those in dogs in Guangdong (6.9%) [19], dogs in Henan (3.8%) [20], dogs in pet markets in Guangzhou (3.2%) [21], companion dogs in Shanghai (8.0%) [22] and Beijing (4.9%) [10], pet dogs in Yunnan (4.6%) [23] and Xinjiang (5.3%) [24], and farm dogs in Heilongjiang (2.2%) [11]. The prevalence of *Cryptosporidium* spp. was also lower than those in previous studies in stray dogs (10.8%) [25]; dogs in Sichuan (11.3%) [26]; dogs in pet market in Guangzhou (3.1%) [21]; dogs in Guangdong (9.4%) [19]; pet dogs in Yunnan (8.0%) [23]; dogs in Beijing (12.8%) [10], Shanghai (8.0%) [22] and Xinjiang (5.3%) [24]; and farm dogs in Heilongjiang (4.5%) [11]. The prevalence rates of *Cryptosporidium* spp. and *G. duodenalis* were lower than those in other reports but higher than those of the other parasites in this study. There seems to be a viable explanation for the higher prevalence; the deworming drug praziquantel that targets tapeworms has no effect on *Cryptosporidium* spp. and *G. duodenalis*. These two protozoans are common in animals and may cause clinical symptoms such as diarrhoea. Therefore, protozoan infections are often occult and consequently are not treated. However, we should pay attention to the public health significance of transmission between dogs and humans, indicating the existence of a zoonotic cycle in the environments, especially rural environments.

Limitations of the current study include uncertainty about the parasite developmental stages in dogs and the ages of the dogs from which the environmental faecal samples were collected. The faecal samples were stored for many weeks in a low-temperature refrigerator in the laboratory prior to genomic DNA extraction and PCR detection. Additionally, only one faecal sample was collected from each dog, and each sample was tested only once. Despite these limitations, the data in the current study highlight the health risks to both humans (herdsmen and pet owners) and dogs (farm dogs and companion dogs) from environmental contamination. It is advised that relevant persons seek guidance from veterinarians about measures to reduce parasitic infections.

The current study demonstrated that zoonotic gastrointestinal parasites were present in dogs in Qinghai, China. These parasites may pose a significant risk to public health, and deworming programmes for dogs should continue to be implemented. Additional preventive measures include dog management and faeces collection and prevention of dog faecal contamination in soil and water environments.

## Figures and Tables

**Table 1:** Intestinal parasites detected by PCR analysis in dogs from urban and rural environments in Qinghai Province.

Parasites	Urban Environments				Rural Environments	
	Chengdong (*n* = 142)	Chengxi (*n* = 129)	Chengnan (*n* = 151)	Chengbei (*n* = 155)	Gangcha (*n* = 35)	Guinan (*n* = 70)
*Echinococcus multilocularis*	0	0	0	0	0	0
*Echinococcus granulosus*	0	0	0	0	0	0
*Echinococcus shiquicus*	0	0	0	0	0	2 (2/70, 2.86%)
*Taenia hydatigena*	1 (1/142, 0.70%)	0	2 (2/151, 1.32%)	1 (1/155, 0.65%)	1 (1/35, 2.86%)	2 (2/70, 2.86%)
*Taenia multiceps*	1 (1/142, 0.70%)	0	1 (1/151, 0.66%)	0	1 (1/35, 2.86%)	1 (1/70, 1.43%)
*Dipylidium caninum*	0	0	0	1 (1/155, 0.65%)	0	1 (1/70, 1.43%)
*Taenia pisiformis*	0	0	0	0	1 (1/35, 2.86%)	0
*Mesocestoides lineatus*	0	0	0	0	0	1 (1/70, 1.43%)
*Trichuris vulpis*	0	0	0	0	1 (1/35, 2.86%)	0
*Toxocara canis*	1 (1/142, 0.70%)	0	1 (1/151, 0.66%)	1 (1/155, 0.65%)	0	1 (1/70, 1.43%)
*Toxascaris leonine*	0	0	0	0	0	0
*Spirocerca lupi*	0	0	0	0	0	0
*Clonorhis sinensis*	0	0	0	0	0	0
*Spirometra mansoni*	0	0	0	0	0	0
*Strongyloides* spp.	0	0	0	0	0	0
*Ancylostoma* spp.	0	0	0	0	0	1 (1/70, 1.43%)
*Giardia duodenalis*	2 (2/142, 1.41%)	1 (1/129, 0.78%)	1 (1/151, 0.66%)	2 (2/155, 1.29%)	1 (1/35, 2.86%)	1 (1/70, 1.43%)
*Cryptosporidium* spp.	3 (3/142, 2.11%)	1 (1/129, 0.78%)	2 (2/151, 1.32%)	1 (1/155, 0.65%)	1 (1/35, 2.86%)	1 (1/70, 1.43%)
*Cystoisospora* spp.	0	0	0	0	0	0
*Neospora* spp.	0	0	0	0	0	0
Total helminths	3 (3/142, 2.11%)	0	4 (4/151, 2.65%)	3 (3/155, 1.94%)	4 (4/35, 11.43%)	9 (9/70, 12.86%)
Total protozoans	5 (5/142, 3.52%)	2 (2/129, 1.55%)	3 (3/151, 1.99%)	3 (3/155, 1.94%)	2 (2/35, 5.71%)	2 (2/70, 2.86%)
Total parasite infections	8 (8/142, 5.63%)	2 (2/129, 1.55%)	7 (7/151, 4.64%)	6 (6/155, 3.87%)	6 (6/35, 17.14%)	11 (11/70, 15.71%)

**Table 2:** Detection of intestinal parasites by Telemann coprological analysis.

Parasites	Urban Environments (577)		Rural Environments (105)		Total (682)	
	*n*	% (95% CI)	*n*	% (95% CI)	*n*	% (95% CI)
*Echinococcus shiquicus*	0	NA	2	1.90 (0.52–6.68)	2	0.29 (0.08–1.06)
*Taenia hydatigena*	4	0.69 (0.27–1.77)	3	2.86 (0.98–8.07)	7	1.03 (0.50–2.10)
*Taenia multiceps*	2	0.35 (0.10–1.26)	2	1.90 (0.52–6.68)	4	0.59 (0.23–1.50)
*Dipylidium caninum*	1	0.17 (0.03–0.98)	1	0.95 (0.17–5.20)	2	0.29 (0.08–1.06)
*Taenia pisiformis*	0	NA	1	0.95 (0.17–5.20)	1	0.15 (0.03–0.83)
*Mesocestoides lineatus*	0	NA	1	0.95 (0.17–5.20)	1	0.15 (0.03–0.83)
*Trichuris vulpis*	0	NA	1	0.95 (0.17–5.20)	1	0.15 (0.03–0.83)
*Toxocara canis*	3	0.52 (0.18–1.52)	1	0.95 (0.17–5.20)	4	0.59 (0.23–1.50)
*Ancylostoma* spp.	0	NA	1	0.95 (0.17–5.20)	1	0.15 (0.03–0.83)
*Giardia duodenalis*	6	1.04 (0.48–2.25)	2	1.90 (0.52–6.68)	8	1.17 (0.60–2.30)
*Cryptosporidium* spp.	7	1.21 (0.59–2.48)	2	1.90 (0.52–6.68)	9	1.32 (0.70–2.49)
Total helminths	10	1.73 (0.94–3.16)	13	12.38 (7.38–20.04)	23	3.37 (2.26–5.01)
Total protozoa	13	2.25 (1.32–3.82)	4	3.81 (1.49–9.39)	17	2.49 (1.56–4.00)
Total parasite infection	23	3.99 (2.67–5.91)	17	16.19 (10.36–24.41)	40	5.87 (4.34–7.89)

## Data Availability

Not applicable.

## Supplementary Materials

The following supporting information can be downloaded at: https://www.mdpi.com/article/10.3390/pathogens11111240/s1, Table S1: Primers used for parasites detection [27,28,29,30,31,32,33,34,35,36,37,38,39,40,41,42,43].
