# Supplementary material for: Prevalence of Intestinal Parasites in Dog Faecal Samples from Public Environments in Qinghai Province, China
Source: Pathogens. 2022 Oct 26;11(11):1240. doi: 10.3390/pathogens11111240 (PMC9696243; doi:10.3390/pathogens11111240)
Supplement: Supplementary file 1 [file pathogens-11-01240-s001.zip › SM Table 1 Primers used for parasites detection.pdf]

Table 1 Primers used for parasites detection

Parasite	Primer sequence	Tm	Product (bp)	References
Echinococcus multilocularis	F: 5'-CATTAATTATGGATGTTTCC-3'	55℃	584	[1]
	R: 5'-GGAAATACCCCACTATCC-3'			
Echinococcus granulosus	F: 5'-GGTTTTATCGGTATGTTGGTGTTAGTG-3'	55℃	219	[1]
	R: 5'-CATTTCTTGAAGTTAACAGCATCACG-3'			
Echinococcus shiquicus	F: 5'-GCTTTAAGTGCGTGACTTTTAATCCC-3'	55℃	496	[1]
	R: 5'-CATCAAAACCAGCACTAATACTCA-3'			
Taenia hydatigena	F: 5'-AGTTCCATATTATTTACAGTTTTGTTATTAC-3'	53℃	618	[2]
	R: 5'-TAACATAATACTTGAAGACACCCCCA-3'			
Taenia multiceps	F: 5'-GTTGTTGATGTGGCTTAAGTTTTTGTGT-3'	54℃	~420	[2]
	R: 5'-TCTATAAAATAAACACATACACAACAATCCT-3'			
Dipylidium caninum	F: 5'-CTATTGATTGCGTTTATTGTTTTGTGT-3'	52℃	218	[2]
	R: 5'-GAAAAGAAATCAAATACAGTTAAACGGT-3'			
Taenia pisiformis	F: 5'-TGTGGGAAGGTTTAGGTGAATCAT-3'	56℃	314	[2]
	R: 5'-GTTAACATCAATATCTTCTAGCTCTGACACT-3'			
Mesocestoides lineatus	F: 5'-TTAAGATATATGTGGTACAGGATTAGATACCC-3'	58℃	~370	[3]

	R: 5'-AACCGAGGGTGACGGGCGGTGTGTACC-3'			
Trichuris vulpis	F: 5'-TCTTGATTCAGTGGGTAGTGG-3'	53℃	399	[4]
	R: 5'-CTTACTGGGAATTCCTCGTTC-3'			
Toxocara canis	F: 5'-GATTTTACCTGCTTTTGGTATTATTAG-3'	53℃	426	[5]
	R: 5'-CCAAAGACAGCACCCAAACT-3'			
Toxascaris leonine	F: 5'-GTAGGTGAACCTGCGGAAGGATCATT-3'	55℃	953	[6]
	R: 5'-TTAGTTTCTTTTCCTCCGCT-3'			
Spirocerca lupi	F: 5'-TGATTGGTGGTTTTGGTAA-3'	50℃	689	[7]
	R: 5'-ATAAGTACGAGTATCAATATC-3'			
Clonorhis sinensis	F: 5'-TTCTTGAGTTGGCTTCCT-3'	50℃	527	[8]
	R: 5'-CCTCAGCAACATAACCAC-3'			
Spirometra mansoni	F: 5'-CACCGAAGCCTGCGGTA-3'	53℃	179	[9]
	R: 5'-GAAGGTCGACCTGGTGAA-3'			
Strongyloides spp.	F: 5'-TTTGATCCTAGTTCTGGTGGTAATCC-3'	56℃	269	[10]
	R: 5'-GTAGCAGCAGTAAAATAAGCACGAGA-3'			
Ancylostoma spp.	F: 5'-CGTGCTAGTCTTCAGGACTTTG-3'	54℃	~540	[11]
	R: 5'-CGGGAATTGCTATAAGCAAGTGC-3'			

Giardia duodenalis	1F: 5'-AAGCCCGACGACCTCACCCGCAGTGC-3'	55°C	753	[12,13]
1st	1R: 5'-GAGGCCGCCCTGGATCTTCGAGACGAC-3'			
2end	2F: 5'-GAACGAACGAGATCGAGGTCCG-3'	55°C	515	
	2R: 5'- CTCGACGAGCTTCGTGTT-3'			
Cryptosporidium spp.	F2: 5'-GACATATCATTCAAGTTTCTGACC-3'	58°C	763	[14,15]
1st	R2: 5'-CTGAAGGAGTAAGGAACAACC-3'			
2end	F1: 5'-CCTATCAGCTTTAGACGGTAGG-3'	58°C	~585	
	R1: 5'-TCTAAGAATTTCACCTCTGACTG-3'			
Cystoisospora spp.	F: 5'-GATCATTCACACGTGGCCCTTG-3'	58°C	~440	[16]
	R: 5'-GACGACGTCCAAATCCACAGAGC-3'			
Neospora spp.	F: 5'-CCCAGTGCGTCCAATCCTGTAAC-3'	59°C	~340	[17]
	R: 5'-CTCGCCAGTCAACCTACGTCTTCT-3'			

References

1. Liu C. N.; Lou, Z. Z.; Li, L.; Yan, H. B.; Blair, D.; Lei, M. T.; Cai, J. Z.; Fan, Y. L.; Li, J. Q.; Fu, B. Q.; et al. Discrimination between *E. granulosus* sensu stricto, *E. multilocularis* and *E. shiquicus* Using a Multiplex PCR Assay. *PLoS. Negl. Trop. Dis.* **2015**, *9*, e0004084.
2. Zhu G. Q.; Li, L.; Ohiolei, J. A.; Wu, Y. T.; Li, W. H.; Zhang, N. Z.; Fu, B. Q.; Yan, H. B.; Jia, W. Z. A multiplex PCR assay for the simultaneous detection of *Taenia hydatigena*, *T. multiceps*, *T. pisiformis*, and *Dipylidium caninum* infections. *BMC Infect. Dis.* **2019**, *19*,

854.
3. Wirtherle N.; Wiemann, A.; Ottenjann, M.; Linzmann, H.; van der Grinten, E.; Kohn, B.; Gruber, A. D.; Clausen, P. H. First case of canine peritoneal larval cestodosis caused by *Mesocestoides lineatus* in Germany. *Parasitol. Int.* **2007**, *56*, 317-320.
4. Htun L. L.; Rein, S. T.; Win, S. Y.; Soe, N. C.; Thein, S. S.; Khaing, Y.; Thaw, Y. N.; Chel, H. M.; Hmoon, M. M.; Bawm, S. Occurrence of gastrointestinal helminths and the first molecular detection of *Ancylostoma ceylanicum*, *Trichuris trichiura*, and *Trichuris vulpis* in dogs in Myanmar. *Parasitol. Res.* **2021**, *120*, 3619-3624.
5. Fava N. M. N.; Cury, M. C.; Santos, H. A.; Takeuchi-Storm, N.; Strube, C.; Zhu, X. Q.; Taira, K.; Odoevskaya, I.; Panovag, O.; Mateus, T. L.; et al. Phylogenetic relationships among *Toxocara* spp. and *Toxascaris* sp. from different regions of the world. *Vet. Parasitol.* **2020**, *282*, 109133.
6. Li M. W.; Lin, R. Q.; Chen, H. H.; Sani, R. A.; Song, H. Q.; Zhu, X. Q. PCR tools for the verification of the specific identity of ascaridoid nematodes from dogs and cats. *Mol. Cell Probes* **2007**, *21*, 349-354.
7. Mohtasebi S.; Teimouri, A.; Abbaszadeh Afshar, M. J.; Mobedi, I.; Abbasian, H.; Totonchian, N.; Mowlavi, G. First report of *Spirocerca lupi* larva in dung beetles (*Scarabaeus armeniacus*) in the central region of Iran: A morphological and molecular identification. *Comp. Immunol. Microbiol. Infect. Dis.* **2021**, *77*, 101671.
8. Yang Y.; Li, M.; Pan, C.; Yang, Y.; Chen, X.; Yao, C.; Du, A. A duplex PCR for the simultaneous detection of *Fasciola hepatica* and *Clonorchis sinensis*. *Vet. Parasitol.* **2018**, *259*, 1-5.
9. Meng Y.; Kuang, Z.; Liao, L.; Ma, Y.; Wang, X. Case Report: Morphologic and Genetic Identification of Cerebral Sparganosis. *Am. J. Trop.*

Med. Hyg. **2019**, *101*, 1174-1176.

10. Beknazarova M.; Barratt, J. L. N.; Bradbury, R. S.; Lane, M.; Whiley, H.; Ross, K. Detection of classic and cryptic *Strongyloides* genotypes by deep amplicon sequencing: A preliminary survey of dog and human specimens collected from remote Australian communities. *PLoS Negl. Trop. Dis.* **2019**, *13*, e0007241.
11. Kladkempetch D.; Tangtrongsup, S.; Tiwananthagorn, S. Ancylostoma ceylanicum: The Neglected Zoonotic Parasite of Community Dogs in Thailand and Its Genetic Diversity among Asian Countries. *Animals (Basel)* **2020**, *10*, 2154.
12. Caccio S. M.; De Giacomo, M.; Pozio, E. Sequence analysis of the beta-giardin gene and development of a polymerase chain reaction-restriction fragment length polymorphism assay to genotype *Giardia duodenalis* cysts from human faecal samples. *Int. J. Parasitol.* **2002**, *32*, 1023-1030.
13. Li X.; Zhang, X.; Jian, Y.; Wang, G.; Ma, L.; Schou, C.; Karanis, P. Detection of *Cryptosporidium* oocysts and *Giardia* cysts in vegetables from street markets from the Qinghai Tibetan Plateau Area in China. *Parasitol. Res.* **2020**, *119*, 1847-1855.
14. Jian Y.; Zhang, X.; Li, X.; Schou, C.; Charalambidou, I.; Ma, L.; Karanis, P. Occurrence of *Cryptosporidium* and *Giardia* in wild birds from Qinghai Lake on the Qinghai-Tibetan Plateau, China. *Parasitol. Res.* **2021**, *120*, 615-628.
15. Ryan U.; Xiao, L.; Read, C.; Zhou, L.; Lal, A. A.; Pavlasek, I. Identification of novel *Cryptosporidium* genotypes from the Czech Republic. *Appl. Environ. Microbiol.* **2003**, *69*, 4302-4307.
16. Samarasinghe B.; Johnson, J.; Ryan, U. Phylogenetic analysis of *Cystoisospora* species at the rRNA ITS1 locus and development of a PCR-RFLP assay. *Exp. Parasitol.* **2008**, *118*, 592-595.

17. Nardoni S.; Poli, A.; Varvaro, I.; Rocchigiani, G.; Ceccherelli, R.; Mancianti, F. Detection of *Neospora Caninum* DNA in Wild Birds from Italy. *Pathogens* **2019**, *8*, 202.
